# The effects of sodium bicarbonate ingestion on swimming interval performance in trained competitive swimmers

**DOI:** 10.1007/s00421-023-05192-6

**Published:** 2023-04-07

**Authors:** L. A. Gough, J. W. Newbury, M. Price

**Affiliations:** 1 Human Performance and Health Research Group, Centre for Life and Sport Sciences (CLaSS), Birmingham City University, Birmingham, B15 3TN UK; 2 Centre for Sport, Exercise and Life Sciences, Coventry University, Coventry, CV1 2DS UK

**Keywords:** Buffering, Supplements, High-performance, Training, Alkalosis

## Abstract

The use of sodium bicarbonate (NaHCO_3_) supplementation to improve repeated high-intensity performance is recommended; however, most swimming performance studies examine time trial efforts rather than repeated swims with interspersed recovery that are more indicative of training sessions. The aim of this study, therefore, was to investigate the effects of 0.3 g.kg^−1^ BM NaHCO_3_ supplementation on sprint interval swimming (8 × 50 m) in regionally trained swimmers. Fourteen regionally competitive male swimmers (body mass (BM): 73 ± 8 kg) volunteered for this double-blind, randomised, crossover designed study. Each participant was asked to swim 8 × 50 m (front crawl) at a maximum intensity from a diving block, interspersed with 50 m active recovery swimming. After one familiarisation trial, this was repeated on two separate occasions whereby participants ingested either 0.3 g.kg^−1^ BM NaHCO_3_ or 0.05 g.kg^−1^ BM sodium chloride (placebo) in solution 60 min prior to exercise. Whilst there were no differences in time to complete between sprints 1–4 (*p* > 0.05), improvements were observed in sprint 5 (*p* = 0.011; ES = 0.26), 6 (*p* = 0.014; ES = 0.39), 7 (*p* = 0.005; ES = 0.60), and 8 (*p* = 0.004; ES = 0.79). Following NaHCO_3_ supplementation, pH was greater at 60 min (*p* < 0.001; ES = 3.09), whilst HCO_3_^−^ was greater at 60 min (*p* < 0.001; ES = 3.23) and post-exercise (*p* = 0.016; ES = 0.53) compared to placebo. These findings suggest NaHCO_3_ supplementation can improve the latter stages of sprint interval swimming performance, which is likely due to the augmentation of pH and HCO_3_^−^ prior to exercise and the subsequent increase in buffering capacity during exercise.

## Introduction

Sodium bicarbonate (NaHCO_3_) is a known legal ergogenic aid that was recommended in the most recent International Olympics Committee (IOC) consensus statement on dietary supplements and the high-performance athlete (Maughan et al. 2018). Most research in swimming opts for 0.3 g.kg^− 1^ BM as this typically achieves the balance between performance enhancement and the management of gastrointestinal (GI) discomfort (Gough et al. 2017a; Newbury et al. 2021; McNaughton et al. 2019). The mechanism to explain the performance effects following NaHCO_3_ supplementation is related to the control of ion fluxes (e.g. hydrogen ions [H^+^]) during high-intensity exercise (McNaughton et al. 2016, 2019). Whilst a topic of contention (Westerblad 2016), exponential H^+^ accumulation is suggested to contribute to fatigue by reducing calcium ion (Ca^2+^) sensitivity and cross-bridge binding, disrupting glycolytic enzyme activity, and the excitation–contraction coupling during high-intensity exercise (Fitts 2008; Spriet et al. 1987). The ingestion of NaHCO_3_ can mitigate the exponential rises in H^+^ during high-intensity exercise, by increasing the flux of H^+^ from active musculature to the blood to be buffered (Bishop et al. 2004). Such mechanisms can potentially be beneficial to swimming due to the high-intensity nature of training and competitive events (Pyne and Sharp 2014).

Ergogenic benefits of NaHCO_3_ supplementation on interval swimming have been mixed within adult cohorts. Gao et al. (1988) reported a significant improvement compared to a placebo in the 4th and 5th sprint of a 5 × 100 yd (91 m) repetitions with 2 min passive rest in-between efforts following ingestion of 2.9 mmol.l^–1^ NaHCO_3_ 60 min prior to exercise. The distance employed (91 m) is not common in swimming, however, whereby a 25, 50 or 100 m distance would be more replicable of competitive swimming. In contrast, Campos et al. (2012) reported no significant impact of NaHCO_3_ ingestion during 6 × 100 m maximal swimming bouts (6 min passive rest in-between), whereby completion times were near identical compared to the placebo. In the latter study, there were no measures for acid base balance (e.g. pH, HCO_3_^−^) and, therefore, it is difficult to debate why no performance effects were observed. However, the authors did opt for capsule ingestion 60 min prior to exercise and it is therefore unlikely HCO_3_^–^ had reached a suitable level to elicit ergogenic effects, as research suggests this peak is around ~ 120 min (Newbury et al. 2021; Jones 2016). Whilst these findings may suggest NaHCO_3_ supplementation is not ergogenic for interval swimming performance, if appropriate methodologies were employed a performance benefit may be seen (e.g., dose timing and exercise protocol). Due to the equivocal nature of the performance benefit to interval swimming following NaHCO_3_, further research is warranted.

One limitation of the work to date is passive rest periods between intervals have been employed. It is intuitive to suggest that active recovery periods could lead to greater acid base balance recovery, and clearance of H^+^ (Dodd et al. 1984), and with the addition of NaHCO_3_, an even greater recovery and potentially improved exercise performance (Siegler et al. 2008). Specifically, Siegler et al. (2008) reported using a 15 min post-exercise active recovery increased the recovery of HCO_3_^–^ compared to a passive recovery following NaHCO_3_ supplementation. The authors reported that recovery of acid base balance (i.e. pH and HCO_3_^−^) recovered at a faster rate in the active vs. passive recovery, and led to a small, non-significant performance improvement compared to passive recovery during 3 × 30 s cycling sprints. Importantly, after the first sprint, HCO_3_^–^ was approximately 3 mmol.l^−1^ greater following NaHCO_3_ ingestion at 180 s recovery following the first 30 s sprint, with similar effects seen in sprint 2 and 3. Based on these findings, it is plausible that if a longer interval set (e.g. 8 × 50 m) was used the accumulative recovery benefits of NaHCO_3_ supplementation and active recovery may lead to greater performance benefits. Equally, longer sets would be more reflective of a typical training set within a cohort of highly trained swimmers (Pollock et al. 2019). To date, however, this has not been explored in the context of interval swimming performance.

If NaHCO_3_ supplementation can improve interval swimming there is potential for enhanced adaptation to training, based on findings related to PGC-1α and Heat Shock Protein 72 (HSP72) (Peart et al. 2013; Percival et al. 2015). Indeed, Peart et al. (2013) reported 4 h post a 4 min ‘all-out’ cycling bout, NaHCO_3_ supplementation attenuated the stress response, as HSP72 expression was lower compared to the placebo at 30 min, 1 h, and 2 h post-exercise compared to the placebo (average increase from pre–post-exercise, placebo = 42% vs. experimental = 5%). These findings suggest that the stress response was attenuated using NaHCO_3_/alkalosis, and this might allow for a greater level of recovery between training bouts. However, this study was in cycling and featured a single ‘all-out’ exercise bout; therefore, it is unknown how this could be applied to swimming training. Moreover, Percival et al. (2015) reported PGC-1α mRNA expression was greater (~ 28%) 3 h post-exercise following NaHCO_3_ supplementation versus placebo, after completion of a HIIT protocol (10 × 60 s cycling sprints at 90% VO_2max_). As PGC-1α is a well-known transcriptional coactivator of several genes required for mitochondrial biogenesis, these increases following NaHCO_3_ supplementation might lead to greater adaptation.

It is unknown, however, if improvements can be seen during interval swimming in a highly trained cohort or if long-term supplementation can improve training adaptation. Evidence from studies using NaHCO_3_ as a training aid is mixed, although few exist in cycling. Specifically, Siegler et al. (2018) reported no effect of NaHCO_3_ on resistance training metrics (e.g. maximal voluntary torque; MVT, rate of torque development; RTD) over 10 weeks. Whilst Bishop et al. (Bishop et al. 2004) reported significant improvements in TTE following NaHCO_3_ compared to a placebo following five training sessions per week for 5 weeks, although this was in mice. It is plausible that if improvements in exercise performance are observed in this cohort NaHCO_3_ supplementation could be an important training aid to improve training performance of similar intensity and volume, which might then lead to greater adaptation. Based on the premise that NaHCO_3_ could improve interval training swimming performance, the purpose of this study was to investigate the effects of 0.3 g.kg^−1^ BM NaHCO_3_ supplementation in a highly trained cohort on 8 × 50 m interval swimming performance interspersed with an active recovery.

## Methods

### Participants

Fourteen trained male competitive swimmers (BM: 73 ± 8 kg) participated in this double-blind, randomised, crossover study. Randomisation was completed using a 4 × 4 block design (NaHCO_3_ first treatment for *n* = 6, placebo first treatment for *n* = 8) accounting for *n* = 16, however, *n* = 2 dropped out due to time commitments. Participants met the category of tier two (‘trained/developmental’) using the classifications provided by McKay et al. (2022). All participants were recruited through a local nationally ranked swimming club’s squads where ages varied from 17 to 22 years old (19 ± 2 years). Participants were trained swimmers that swam 7–10 times a week accumulating to 15–20 h a week. Land-based training was also undertaken 3 to 5 times a week primarily consisting of resistance training. Participants competed in up to 12 competitions a year, which included national/international standard competitions that required qualification through the British Swimming Association standards. Swimmers were mixed in terms of stroke preference and participated across middle (100–400 m) and long distance (> 400 m) primarily (*n* = 11), although some (*n* = 3) did also do sprint (50–100 m). All participants were informed of the procedures and possible side effects of the study through written informed consent; parental consent was given for participants under the age of 18. This study received institutional ethical approval (Newbury/7595/HELSFAEC).

### Exercise protocol

All participants completed the exercise protocol during their normal training time and at the same time of day (± 1 h) (range: 5–8 pm). Following a familiarisation trial that entailed completing the interval sets, the experimental protocol was repeated on two occasions 1 week apart. Each participant was asked to swim 50 m (front crawl), from a competitive diving block, at their maximum effort and repeat this 8 times. After each 50 m sprint, participants undertook a 50 m active recovery swim requiring them to swim at their normal warm–up pace. Each exercise bout was run off a 5 min base, which left the participants with approximately 3 min passive recovery. Although similar exercise protocols have utilised passive recovery (Gao et al. 1988; Zajac et al. 2009), the current protocol replicated a typical training session for the squad. The exercise protocol was designed following consultation with both regional and national coaches in the United Kingdom who had oversight of high-performance swim programs.

On both testing sessions participants ingested either a solution of NaHCO_3_ (0.3 g.kg^−1^ BM) or sodium chloride (0.05 g.kg^−1^ BM) mixed with low calorie orange squash (0.2 ml.kg^−1^·bm) and tap water (0.3 ml.kg^−1^ BM). This dose was selected as it has been shown to produce ergogenic effects whilst balancing GI discomfort compared to lower or higher doses (McNaughton et al. 2016). Treatments were double blind and administered in a block randomised order. The sodium chloride dosage was used to replicate the taste of sodium bicarbonate with participants agreeing to similar tastes (Price et al. 2003), and no participants in the current study could identify which treatment they had received upon verbal questioning following each experimental trial. Each solution was consumed 60 min prior to testing to allow time for absorption into the blood and for an adequate warm-up period. The warm-up was approximately 2000 m as determined by the head coach and replicated a typical squad session. Participants maintained a low to moderate intensity pace that kept their heart rate between 130 and 160 bts·min^−1^. All warm-ups were consistent between participants.

Fingertip capillary blood samples were taken at three time points; baseline (pre-ingestion), 60 min post-NaHCO_3_ ingestion, and immediately post-exercise. A 5 µL sample was taken for blood lactate concentration ([BLa]) and was analysed using a hand-held device (Lactate Pro LT-1710). Blood pH and HCO_3_^−^ concentrations were analysed using a reliable blood gas analyser (Radiometer, ABL5, Copenhagen, Denmark) after taking a 70 µL blood sample (Gough et al. 2017; Stadlbauer et al. 2011). The blood gas analyser was calibrated prior to use as per the manufacturer instructions and all quality control checks passed prior to use. Heart rate (HR; Polar beat heart rate monitor), ratings of perceived (RPE) (Borg scale; 6–20; Borg 1982) and sprint time were recorded after every 50 m sprint by experienced coaches. Participants were also asked to wear their usual training swimwear for each experimental trial, as well as only consuming either water or squash during each test. Assessments for GI were conducted every 10 min from ingestion to pre-exercise using visual analogue scales (VAS), as per previous research (Gough et al. 2017a). Symptoms for nausea, flatulence, stomach cramp, bowel urgency, diarrhoea, vomiting, stomach bloating, belching, stomachache, headache, and thirst were collected.

### Statistical analysis

Data were initially checked for normality using Shapiro–Wilk and standard geographical methods (e.g. Q–Q plots and Histograms). For time to complete each 50 m set, a two-way (treatment: NaHCO_3_ or PLA x each 50 m set) repeated measures Analysis of Variance (ANOVA) was conducted, using a Bonferroni correction. Homogeneity of variance/sphericity were analysed using Mauchly tests and any violations were corrected if required (e.g. via Greenhouse–Geisser or Huynh–Feldt adjustments). This test was also used for blood variables (pH, HCO_3_^−^ and [BLa]), HR, and RPE. For any ANOVA interaction or main effects, partial eta squared effect size is reported (*ɳ*^2^). A paired samples *t* test was used for aggregated GI discomfort, and aggregated swimming performance (sum of 8 × 50 m in seconds). To determine individual changes in time to complete, the Smallest Worthwhile Change (SWC) statistic was used (0.3* between-subject standard deviation; SD) for each interval sprint (Paton and Hopkins 2006). Hedge’s *g* effect size was calculated (due to n < 20) by dividing the mean difference between trials by the pooled SD (Lakens 2013), and interpretation was as follows: trivial (≤ 0.2), small (0.2–0.49), moderate (0.5–0.79) or large (≥ 0.8) (Cohen 1988). Data are presented as Mean ± SD (unless stated otherwise). Statistical significance was set at *p* < 0.05 and all statistical data were analysed using SPSS software version 28 (IBM, Chicago, IL, USA).

## Results

### Swimming performance

**Fig. 1:**
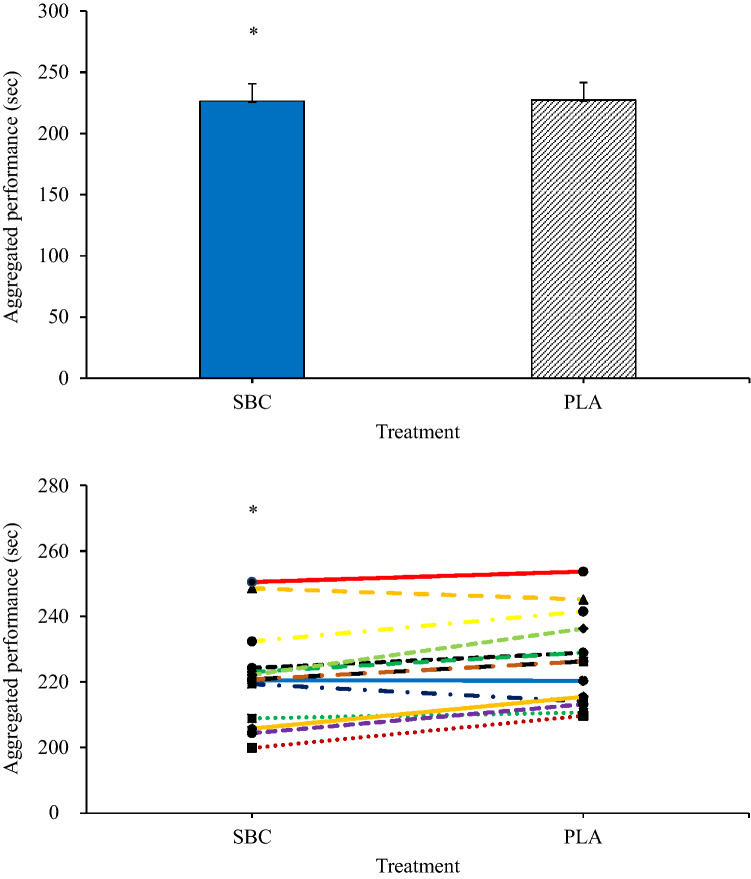
Aggregated performance following sodium bicarbonate (SBC) ingestion or placebo. Group mean data (top) and individual data (bottom). *Denotes significant difference between (*p* < 0.05). NaHCO_3_ = 226.5 ± 14.0 s, PLA = 227.4 ± 14.3 s

Performance was improved by NaHCO_3_ ingestion during the interval swimming protocol (*p* = 0.005, *ɳ*^2^ = 0.301). No improvements were observed between sprints 1–4 (*p* > 0.05), however, improvements were observed in sprint 5 (mean difference = – 0.5 s; *p* = 0.011; ES = 0.26), 6 (mean difference = – 0.8 s; *p* = 0.014; ES = 0.39), 7 (mean difference = – 1.0 s; *p* = 0.005; ES = 0.6), and 8 (mean difference = – 1.4 s; *p* = 0.004; ES = 0.79) (Table 1). Aggregated performance (sum of 8 intervals) was faster following NaHCO_3_ ingestion compared to placebo (NaHCO_3_ = 226.5 ± 14.0 s, PLA = 227.4 ± 14.3 s; *p* = 0.008, ES = 0.06; Fig. 1). Following NaHCO_3_, the drop off in performance from sets 1–4 was – 0.4 s and – 0.1 s between 5 and 8, whilst the placebo dropped off by – 0.7 s and – 0.9 s, respectively.

**Table 1:** Mean and standard deviation (SD) performance times following placebo (PLA) and sodium bicarbonate (SBC)

Participant number	Interval 1		Interval 2		Interval 3		Interval 4		Interval 5		Interval 6		Interval 7		Interval 8	
	PLA	SBC	PLA	SBC	PLA	SBC	PLA	SBC	PLA	SBC	PLA	SBC	PLA	SBC	PLA	SBC
1	26.8	27.3	27.0	27.4	27.0	27.6	26.9	27.0	26.7	27.3	26.4	27.8	26.6	27.4	26.7	27.5
2	29.9	28.0	28.4	28.0	28.2	27.9	28.4	27.9	28.5	28.4	28.7	27.9	28.5	28.2	28.5	28.0
3	25.3	26.0	25.6	25.8	25.5	25.5	25.6	25.9	26.1	26.1	27.1	26.5	27.8	26.7	27.7	26.6
4	26.6	26.6	27.4	27.2	28.0	27.4	28.8	27.8	29.4	28.4	29.7	28.6	29.5	28.5	29.5	28.5
5	26.7	27.9	26.7	27.2	26.5	27.4	27.4	28.2	28.0	27.8	28.2	27.7	28.4	27.2	28.5	27.2
6	30.7	30.7	30.8	31.1	30.7	31.7	30.7	31.0	30.4	31.0	30.6	30.6	30.5	30.9	30.7	31.6
7	31.4	30.4	31.2	30.1	31.2	30.3	31.5	32.7	33.0	32.1	32.4	32.4	31.4	31.8	31.6	30.8
8	25.4	25.9	26.0	26.3	26.7	26.7	27.4	27.0	27.7	27.1	27.6	27.9	28.0	28.0	28.3	27.8
9	27.8	27.8	27.7	27.4	28.1	27.4	27.9	27.2	28.2	27.8	28.6	27.9	29.0	27.8	29.0	27.5
10	25.2	24.7	25.3	24.5	25.4	24.4	26.5	25.6	26.4	25.8	28.5	26.2	29.1	27.4	29.1	27.2
11	29.2	29.1	28.9	29.1	29.1	29.0	30.1	29.0	30.3	28.7	30.4	29.2	31.0	29.2	32.5	29.1
12	25.0	25.1	26.4	24.7	25.8	25.6	26.9	25.1	27.2	26.5	27.1	25.8	26.9	25.2	27.9	26.4
13	24.5	24.9	24.9	24.8	25.1	25.4	25.5	25.1	26.4	25.2	26.9	24.5	27.5	24.9	28.9	25.1
14	28.8	27.4	28.4	27.1	29.0	28.0	29.4	27.4	29.1	28.0	30.0	28.4	30.1	27.9	31.5	28.0
Mean	27.4	27.3	27.5	27.2	27.6	27.4	28.1	27.6	28.4	27.9*	28.7	28.0*	28.9	27.9*	29.3	28.0*
SD	2.2	1.8	1.9	1.9	1.9	1.9	1.8	2.1	1.9	1.8	1.6	1.9	1.4	1.8	1.6	1.6

*SD* standard deviation

*Denotes significance difference between SBC and PLA (*p* < 0.05)

### Blood variables

**Fig. 2:**
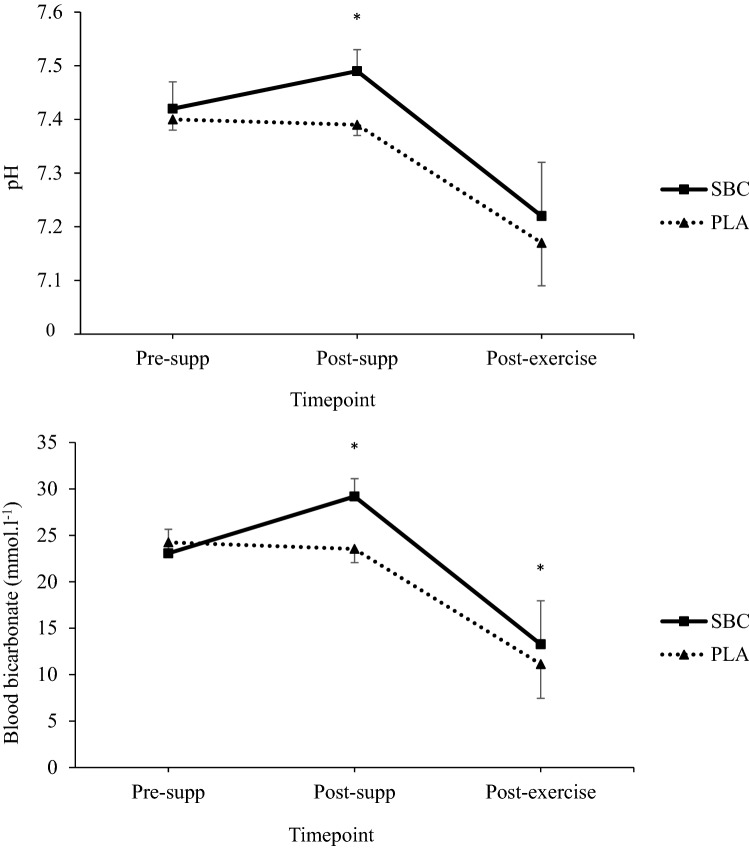
Time course changes in pH (top) and blood bicarbonate (HCO_3_^−^) (bottom) following placebo or sodium bicarbonate (SBC). Some error bars omitted for clarity. *Denotes significant difference (*p* < 0.05)

Following NaHCO_3_ supplementation pH was greater at 60 min (+ 0.07 a.u; *p* < 0.001; ES = 3.09), whilst HCO_3_^−^ was greater at both 60 min (+ 5.7 mmol.l^−1^; *p* < 0.001; ES = 3.23) and post-exercise (+ 2 mmol.l^−1^; *p* = 0.016; ES = 0.53) (Fig. 2). The change in HCO_3_^−^ during exercise was greater following NaHCO_3_ supplementation compared to placebo (+ 3.5 mmol.l^−1^; *p* < 0.001; ES = 0.88; Table 2). Blood lactate concentration was greater following NaHCO_3_ supplementation post-exercise compared to placebo (17.6 ± 4.9 vs. 14.7 ± 3.8 mmol.l^−1^; *p* < 0.001; ES = 0.64). There was a large variation in blood responses from baseline to 60 min following NaHCO_3_ ingestion (Table 2).

**Table 2:** Blood responses following sodium bicarbonate (SBC) supplementation and placebo (PLA)

Participant number	Blood bicarbonate (HCO_3_^−^) baseline – 60 min (post SBC supplementation) (mmol.l^−1^)	HCO_3_^−^ change during exercise (post SBC supplementation) (mmol.l^−1^)	HCO_3_^−^ change during exercise (post PLA supplementation) (mmol.l^−1^)
1	6	17	17
2	10	15	13
3	4	20	12
4	5	22	16
5	1	12	14
6	7	20	16
7	12	7	9
8	5	24	17
9	5	15	11
10	7	17	13
11	7	15	10
12	4	11	7
13	5	14	10
14	7	15	9
Mean	6.1	15.9*	12.4
SD	2.6	4.4	3.2

*SD* standard deviation

*Denotes significant difference (*p* < 0.05)

### Heart rate, GI and RPE

There were no differences in HR at any time point between NaHCO_3_ and placebo (*p* = 0.883, Pƞ^2^ = 0.061). Aggregated GI discomfort was higher for NaHCO_3_ compared to placebo (21 ± 12 vs. 3 ± 1 a.u; *p* = 0.021; ES = 2.05). There were no differences in RPE between NaHCO_3_ and the placebo (*p* = 0.754, Pƞ^2^ = 0.024).

## Discussion

This study aimed to investigate the effects of NaHCO_3_ ingestion on swimming interval performance in trained competitive swimmers. The results suggest NaHCO_3_ ingestion improves swimming interval performance in the latter stages of the set compared to a placebo, which was likely caused by the increase in buffering capacity. However, not all participants improved and GI discomfort was apparent in several swimmers. Based on these findings, NaHCO_3_ ingestion should be tested on an individual basis for suitability. These findings are still important for swimmers nonetheless that are aiming to increase their performance in training and is an effective ergogenic aid. The current study also reports, for the first time, that NaHCO_3_ is a suitable ergogenic aid when an appropriate dose and timing of ingestion is employed, along with an active recovery period between bouts. Lastly, due to the improvement in performance, there is scope for this supplement to improve overall training adaptation; however, further research is warranted to investigate NaHCO_3_ ingestion over a training block to confirm this inference.

The current study corroborates with previous research reporting improvements in interval swimming performance following NaHCO_3_ ingestion (Siegler and Gledall-Sidall 2010; Gao et al. 1988; Zajac et al. 2009). Of note, is the greater training status of participants employed in the current study compared to Siegler and Gledall-Sidall (2010), such that our findings can be applied to those that are at the competitive end of swimming performance. Furthermore, as the current study utilised an active recovery and an interval set of exercise, which is commonly employed by coaches in training (Pollock et al. 2019), our results can be applied directly to improve swimming interval performance. Building upon previous work by Zajac et al. (2009) in adolescent athletes, the current study reports that improvements can be seen in adults. Moreover, as performance improved and similar acid base balance responses were observed in the current study compared to Percival et al. (2015) and Peart et al. (2013), it is plausible that if this strategy was applied chronically greater adaptations to training might be observed through changes in PGC-1α and HSP72. Favourable adaptations in PGC-1α could improve mitochondrial biogenesis, which may increase mitochondrial content and oxidative enzyme activity (Gollnick et al. 1973), particularly following training exercises (i.e. high intensity) like that employed in the current study (MacInnis et al. 2017). As the study was not mechanistic in nature we cannot confirm if these changes were present, however, this opens an area for future research.

As a group, there was an improvement in swim performance during the latter half of the repeated sprints (sprints 5–8). However, the ergogenic effects of NaHCO_3_ were not apparent across all the participants at the same time points. Specifically, 10 of the 14 participants improved above the SWC in sprint interval eight, whereas only 4 improved by sprint interval three. These observations indicate that the ergogenic effects became apparent towards the latter part of the exercise, which was approximately three minutes into the exercise. It can be speculated that those participants not improving performance may have had sub-optimal ingestion strategies. Recently, a time to peak HCO_3_^−^ approach, whereby participants ingest NaHCO_3_ at their respective time to peak HCO_3_^−^ prior to exercise, has been shown to increase the chances of securing an ergogenic benefit (Boegman et al. 2020; Gough et al. 2018). Using this strategy might have allowed the other participants to obtain ergogenic effects, as Boegman et al. (2020) showed this approach was more effective at improving 2000 m TT rowing performance compared to the standardised ingestion strategy (as used in the current study). However, this procedure was not conducted in the current study due to logistical constraints with this highly trained cohort. Future research could, therefore, adopt the time to peak HCO_3_^−^ strategy and compare with a standardised time frame to see if this allows more participants to benefit from NaHCO_3_ supplementation.

The group mean increase in HCO_3_^−^ following NaHCO_3_ ingestion was ~ 6 mmol.l^−1^, demonstrating the study protocol was successful at achieving a level of alkalosis similar to other studies, and the increase purported to improve exercise performance (Carr et al. 2011; Lopes-Silva et al. 2022). It is important to note, however, that not all participants met this threshold and still managed to improve their performance, and vice versa, which subsequently questions whether this ergogenic threshold is required. Following on from previous findings (Gough et al. 2021), both participants six and seven in the current study increased their HCO_3_^−^ from baseline to post-supplementation comfortably over this purported threshold, yet did not improve performance (7 and 12 mmol.l^−1^, respectively). Conversely, participant 12 and 13 increased by 4.3 and 4.8 mmol.l^−1^, respectively, yet improved their performance over the SWC of the test. This evidence suggests that a threshold of change in HCO_3_^−^ is not as clear as first seems, and instead, it is more important to trial different doses to identify the physiologically optimal dose on an individual basis.

Whilst the current study was more applied in nature, it does offer insight into the mechanism of action for NaHCO_3_ ingestion. Indeed, significant pre-exercise increases in pH and HCO_3_^−^ were observed compared to placebo and this infers an increased buffering capacity during exercise. This is also shown by the significantly increased blood lactate, although this could have been due to the increased exercise intensity in the NaHCO_3_ performance, and not a marker of increased buffering capacity. Nonetheless, in agreement with the findings of Bishop et al. (2004), the current study infers the ingestion of NaHCO_3_ increased the movement of H^+^ from intracellular to extracellular compartments, and this could have reduced the level of fatigue in participants. Of note, the change in HCO_3_^−^ during exercise corroborates this notion as this was significantly greater following NaHCO_3_ ingestion from pre- to post-exercise compared to placebo, in line with previous studies (Gough et al. 2018, 2019). Moreover, the current study employed an active recovery between bouts, and this may have heightened the ergogenic effects compared to previous studies using a passive rest and reporting no effect. This is based on the accelerated recovery of acid base balance previously reported by Siegler et al. (2008) following NaHCO_3_ compared to placebo. Notwithstanding, these inferences are restricted to extracellular measurements, and as such, future research more mechanistic in nature could continue to explore this theory of fatigue to elucidate the full mechanisms following NaHCO_3_ ingestion. Equally, future research may include blood measurements during each interval if this is logistically possible.

Applied studies are always open to limitations, and this study presents limitations in the ingestion strategy adopted and the lack of mechanistic insight. Recently, a string of studies (Gough et al. 2017b, 2018, 2019; Boegman et al. 2020) have suggested/evidenced that using an individual time to peak HCO_3_^−^ strategy (mapping ingestion timing to each individual time to peak HCO_3_^−^) improves performance compared to the standardised ingestion strategy in this study. However, it was logistically too difficult to determine individual time to peak HCO_3_^−^, and this approach also has some criticisms in that an ergogenic window might be present following NaHCO_3_ ingestion of up to 2 h; therefore, the individual peak may not be required (Farias de Oliveira et al. 2020). Moreover, it was also observed that a number of swimmers suffered GI discomfort which may limit the practical use of NaHCO_3_ in a real-world setting. Specifically, two participants suffered from diarrhoea that caused disruption to their usual preparation for training. On the other hand, the other participants in the study had general mild-moderate stomachache, belching and bloating. It is important to, therefore, trial NaHCO_3_ on an individual basis; however, for most, it will be tolerable.

## Conclusion

To conclude, this study reports that the ingestion of NaHCO_3_ can improve swimming interval performance reflective of a training bout. Not all participants improved their performance, however, and therefore, this supplement should be trialled on an individual basis. Further research is warranted to explore how NaHCO_3_ could impact training adaptation.

## Data Availability

Data is available upon request to the corresponding author.

## Abbreviations

NaHCO_3_: Sodium bicarbonate
HCO_3_^–^: Bicarbonate
H^+^: Hydrogen ion
pH: Potential hydrogen
Ca^2+^: Calcium
GI: Gastrointestinal discomfort
VAS: Visual analogue scale
HSP72: Heat shock protein 72
ANOVA: Analysis of variance
*ɳ*^2^: Partial eta squared
